# Global Proteomic Analysis Reveals High Light Intensity Adaptation Strategies and Polyhydroxyalkanoate Production in *Rhodospirillum rubrum* Cultivated With Acetate as Carbon Source

**DOI:** 10.3389/fmicb.2020.00464

**Published:** 2020-03-25

**Authors:** Guillaume Bayon-Vicente, Ruddy Wattiez, Baptiste Leroy

**Affiliations:** Laboratory of Proteomics and Microbiology, Research Institute for Biosciences, University of Mons, Mons, Belgium

**Keywords:** purple bacteria, acetate assimilation, photoheterotrophy, photosynthetic metabolism, redox homeostasis, proteomic, volatile fatty acid (VFA)

## Abstract

Purple non-sulfur bacteria (PNSBs) are well known for their metabolic versatility. Among them, *Rhodospirillum rubrum* can assimilate a broad range of carbon sources, including volatile fatty acids (VFAs), such as acetate, propionate or butyrate. These carbon sources are gaining increasing interest in bioindustrial processes since they allow reduction of the production costs. Recently, our lab discovered that, after long term cultivation with acetate as unique carbon source, *Rs. rubrum* got acclimated to this carbon source which resulted in a drastic reduction of the lag phase. This acclimation was characterized by the amplification of the genomic region containing, among others, genes belonging to the ethylmalonyl-CoA (EMC) pathway, which has been demonstrated to be required for acetate assimilation in *Rs. rubrum*. In this paper, we combined bacterial growth analysis with proteomic (SWATH -Sequential Windowed Acquisition of All Theoretical Fragment Ion Mass Spectra-processing) investigation to better understand the bacterial response to a sudden increase of the light intensity. We compared the impact of suddenly increasing light intensity on the WT strain to that on the newly described acetate-competent strain in the presence of acetate. Contrary to what was observed with the WT strain, we observed that the acetate-competent strain was tolerant to the light stress. Proteomic analysis revealed that increasing light intensity had a significant impact on the photosynthetic apparatus, especially in the wild-type strain cultivated in the presence of acetate and low concentration of HCO_3_^–^. This phenomenon was accompanied by a relatively higher abundance of certain stress related proteins. Our results suggested that the production of PHA, but also potentially of branched chain amino acids synthesis, could be part of the mechanism used by *Rs. rubrum* to adapt to the light stress and the redox imbalance it triggered.

## Introduction

*Rhodospirillum rubrum* (*Rs. rubrum*) is a purple non-sulfur bacterium (PNSB) belonging to the α-proteobacteria class and is well known for its metabolic versatility performing either autotrophic or heterotrophic metabolism. *Rs. rubrum* is able to grow using aerobic respiration as well as anoxygenic photosynthesis using light as an energy source (McEwan, 1994). This photosynthetic bacterium can assimilate a broad range of carbon sources, including volatile fatty acids (VFAs). These compounds often result from fermentation processes and are consequently found in wastewater treatment effluents (Bengtsson et al., 2008), activated sludge (Morgan-Sagastume et al., 2011), or brewery waste streams (Shao et al., 2008). The use of VFAs as a carbon source in biotechnological applications using purple bacterial cultures could help to reduce the production costs of high added value compounds such as polyhydroxyalkanoates, proteins or pigments.

It is now well accepted that in organisms lacking isocitrate lyase gene (*icl*^–^) (Kornberg and Krebs, 1957), such as *Rs. rubrum*, an alternative pathway is used for acetate assimilation (i.e., the ethylmalonyl-CoA pathway) (Leroy et al., 2015). In addition to this pathway, the implication of other pathways in acetate photoheterotrophic assimilation has been proposed, and further studies are still needed to attest their possible involvement in acetate assimilation. Among them, our group reported the functions of pyruvate ferredoxin oxidoreductase (PFOR) and glutaryl-CoA dehydrogenase (Leroy et al., 2015). Recently, our lab investigated the acclimation occurring after long-term cultivation (50 generations) of the wild-type strain of *Rs. rubrum* with acetate as the sole carbon source. This acclimation was characterized by the amplification and upregulation of a 60 kb genome fragment corresponding to approximately 120 genes containing genes coding for key enzymes of the ethylmalonyl-CoA pathway [i.e., crotonyl-CoA carboxylase/reductase (Rru_A3063), (2*R*)-ethylmalonyl-CoA mutase (Rru_A3062), and methylsuccinyl-CoA dehydrogenase (Rru_A3064)]. Proteomic analysis revealed a higher abundance of the corresponding enzymes, but also of most of the proteins of the amplified gene cluster. The resulting phenotype showed a drastically reduced lag phase in the presence of acetate (De Meur et al., 2018). Due to the reduced lag phase, this newly adapted strain named “acetate-competent strain” also constitutes a great interest for biotechnological processes. Our hypothesis to explain the observed phenotype is that acclimation reduces the stress occurring during the early phase of growth. This strain thus also represents a very interesting model for studying light stress in *Rs. rubrum.* We demonstrated that the duration of the lag phase is linked with inoculum size, suggesting that high light intensity could be deleterious to diluted cells (Leroy et al., 2015). We proposed that high light intensity, resulting from the dilution of the cells at inoculation, could result in an intracellular imbalance in the cofactor pool. Indeed, in case of sudden light increase, the resulting excess in proton motive force has been shown to increase the reverse flux through NADH dehydrogenase, reducing NAD^+^ to NADH (Herter et al., 1998; Golomysova et al., 2010). Supporting this redox imbalance hypothesis, a reduction in the lag phase could also be obtained using a higher concentration of bicarbonate ions (50 mM) in the medium (De Meur et al., 2018), suggesting that the bicarbonate fixation, which act as an electron sink, helped cells to start growing. Importantly, proteomic data showed lower relative abundance of the Ribulose Bisphosphate Carboxylase/Oxygenase (RuBisCO), key enzyme of the Calvin-Benson-Bassham- (CBB) cycle in the acetate condition compared to the succinate condition, ruling out involvement of CBB cycle in electron sinking in this case. This observation was explained by the fact that the ethylmalonyl-CoA pathway offers another HCO_3_^–^-consuming pathway, probably helping to maintain the redox homeostasis.

In this study, we analyzed the response of *Rs. rubrum* WT and acetate competent strain to sudden increase of light intensity. We characterized this response in various conditions of carbon sources as well as availability of bicarbonate ions, analyzing carbon consumption, growth and global metabolism through proteomic. As PHAs have been proposed as an electron sink in purple bacteria and as they represent an important biotechnological application, we also analyzed the production of PHAs in response to the sudden increase in the light intensity.

## Materials and Methods

### Bacterial Strain, Medium Composition, and Cultivation Conditions

*Rhodospirillum rubrum* S1H (ATCC 25903) wild-type (WT) and acetate-competent strains were cultivated in medium as described previously in Leroy et al. (2015) with a starting OD_680 nm_ = 0.500. The upper gaseous phase was flushed using pure N_2_ and flasks were hermetically sealed. The medium was supplemented with acetate (62.5 mM) or succinate (31.25 mM) as the carbon source and a defined amount of bicarbonate ions (0–3–50 mM). Pre-cultures used for the different experiments were grown in presence of succinate excepted for acetate acclimated strains where pre-cultures were cultivated in presence of acetate in order to avoid the reported deacclimatation of the strain (De Meur et al., 2018). Cultures were subjected to 50 μmol photons/m^2^ s (Sencys; 10 W; 100 lumens; 2,650 K) and incubated with rotary shaking at 180 rpm at 30°C. To perform light stress experiments (see below), this intensity was elevated from 50 μmol photons/m^2^ s to 150 μmol photons/m^2^ s. Growth was monitored by measuring the optical density at 680nm.

### Monitoring of the Carbon Source and Branched Amino Acids (BCAAs) in the Medium

Monitoring of the carbon source concentration was performed as described Leroy et al. (2015). The carbon source concentration was assayed by integrating the carbon source specific peak (RT_acetate_ = 11.27 min; RT_succinate_ = 9.26 min) and based on a standard curve constructed with the corresponding standards.

BCAAs concentration in the culture medium was monitored using the Branched chain amino acid (Leu/Ile/Val) colorimetric assay kit (Biovision^®^, K564) following the manufacturer’s instruction.

### Total Carbohydrate Assay

Total carbohydrates were extracted as described by Fradinho et al. (2013). The resulting carbohydrates were analyzed using a colorimetric reaction as described by Dubois et al. (1956) with minor modifications. Briefly, 10 μL of a phenol:ethanol (80:20, w/v) solution and 1 mL of 100% H_2_SO_4_ were added to 400 μL of filtered supernatant. The mixture was then incubated for 10 min at room temperature. The absorbance of the furfural product was then read at 490 nm and compared to a glucose standardization curve.

### Proteomic Analysis

Bacteria were harvested via centrifugation at 16,000 g at 4°C before and after increasing light intensity, which correspond to OD_680nm_ values of 1.4–1.6 and 2.5–3.0, respectively, and the resulting pellets were subjected to protein extraction. Proteins were extracted as described in Leroy et al. (2015) with minor modifications. Following trypsin digestion, proteins were then purified using the HyperSep^TM^ SpinTip C-18 kit (Thermo Fisher Scientific, United States) following the manufacturer’s instructions. Samples were then subjected to Speed Vac and resuspended in 50 μL of loading buffer consisting of 2% ACN and 0.1% formic acid before being quantified using the Pierce^TM^ Quantitative Colorimetric Peptide Assay, Thermo Fisher Scientific.

Protein identification and quantification were performed following a label-free strategy on a UHPLC HRMS platform (Eksigent 2D ultra-AB SCIEX TripleTOF^TM^ 6600). Peptides (2 μg) were separated on a 15 cm C18 column (3C18-CL-120, eksigent) using a linear acetonitrile (ACN) gradient [5–35% (v/v), in 120 min] in water containing 0.1% formic acid (v/v) at a flow rate of 300 nl.min^–1^. Peptide spectra were acquired in data-dependent (DDA) and data-independent (DIA, SWATH) acquisition modes. The MS/MS library needed for DIA analysis was built using DDA mode and ProteinPilot software (version 4.5, AB Sciex, United States). The algorithm Paragon (version 4.5.0.0, AB Sciex, United States) was used to search the UniProt database restricted to *Rhodospirillum* entries (ATCC 11170 + F11 strains). The search parameters included differential amino acid mass shifts for carbamidomethyl cysteine, oxidized methionine, all biological modifications, amino acid substitutions and missed trypsin cleavage sites.

For SWATH analyses, 100 incremental steps were defined as windows of variable m/z values over a 400–1250 m/z mass range. The MS/MS working time for each window was 50 ms, leading to a duty cycle of 5 s per cycle. The ion chromatogram of the top six fragments of each peptides was extracted, and their area under the curve was integrated. PeakView^®^ software (version 2.1.0.11041, AB Sciex, United States) was used for the SWATH processing of all proteins identified considering an FDR below 1% (as determined by ProteinPilot). The retention time (RT) was recalibrated manually from a group of 15 selected peptides with a RT in the range of 20–100 min. Intensity of peptides were individually normalized based on a summed area of all peptides for each sample. Only proteins quantified with 2 or more peptides were considered. Only fold change higher than 1.5 or lower than 0.66 and having a *p*-value lower than 0.05 were further considered.

All computed data, as well as raw data, have been uploaded on PeptideAtlas and are freely accessible^1^.

### PHA Extraction, Quantitation and Calculation of Production Yield

PHAs were isolated as described in Snell et al. (2002) with some modification. Briefly 500 μL of culture were centrifuged (8000 rpm, 15 min) and stored at −20°C till analyze. PHAs were extracted and methanolysed by resuspending pellets in 500 μL of chloroform and 2 mL of methanolysis solution consisting in UHPLC methanol: concentrated HCl (90:10). The methanolysis solution also includes 0.1 mg/mL of 3-methylbenzoic acid as reaction house standard. Mixture was then incubated at 100°C during 2 h before being cooled down on ice. One milliliter of distilled water was then added and the bottom chloroform part was recovered and analyzed by GC-MS.

### Statistical Analyses

All experiments were performed in five replicates. Concerning proteomic analyses, MarkerView^TM^ 1.2.1 (ABSciex, United States) was used for statistical treatment of the data (i.e., Student’s *t*-test, PCA). The selected proteins were plotted in a generalized heatmap, and protein response groups (PRGs) were defined with a hierarchical cluster dendrogram (Euclidian distance) using the *gplots* RGui package following procedure of Jacquiod et al. (2017). The statistical validity and robustness of the obtained PRGs were tested against a null-model by the Monte-Carlo simulation (10,000 permutations, 95% confidence interval, *p* < 0.05, see Supplementary Figure S1). All graphs were plotted using the software GraphPad Prism (version 6.01, GraphPad Prism Software, United States). The results were reported as the means and standard deviations computed by GraphPad Prism 6.01.

## Results and Discussion

### Bicarbonate Ion Dependency of the Growth of *Rs. rubrum* in the Presence of Acetate

It is well known that the assimilation of acetate, propionate, butyrate or valerate as a sole source of carbon in anaerobic environments is associated with the consumption of HCO_3_^–^ (Rinne et al., 1965; Porter and Merrett, 1972; Ehrenreich and Widdel, 1994; Laguna et al., 2011). In the case of acetate, carbonate ions were shown to drastically reduce the lag phase encountered at the onset of growth with acetate as sole carbon source (De Meur et al., 2018). Not only bicarbonate concentration can help bacterial growth start up, since we also demonstrated that a reduced lag phase could be obtained by inoculating the culture at a high OD_680nm_ (Leroy et al., 2015). These results suggested that the lag phase could be imputed to a redox imbalance due to the increased light intensity when cells are diluted by the inoculation in fresh medium.

**FIGURE 1:**
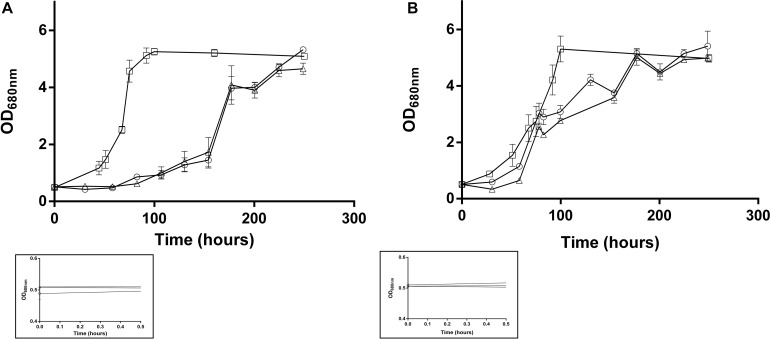
Growth of the *Rs. rubrum* WT **(A)** or acetate-competent **(B)** strains in medium containing different concentration of added bicarbonate ions (0 mM: triangle; 3 mM: circle and 50 mM: square). Framed growth curves represent the early growth phase and the starting inoculum, *n* = 5.

To better understand the assimilation of acetate, *Rs. rubrum* was grown in the presence of acetate and different bicarbonate ion concentrations. As shown in Figure 1A, and similarly to observations reported in Leroy et al. (2015) and De Meur et al. (2018), the wild-type strain was able to grow in “low bicarbonate condition” (i.e., 3 or 0 mM of HCO_3_^–^ added in the culture medium). It has to be noted that, as the anaerobic conditions are obtained in this study by only flushing the gas phase of the culture with nitrogen, residual bicarbonate ions are supposed to be present in the liquid phase of the culture at a level estimated to 1 mM. The culture medium containing 0 and 3 mM of HCO_3_^–^ are thus both considered as “low bicarbonate conditions.” In “low bicarbonate conditions,” growth of the WT was preceded by a particularly long lag phase, which could be drastically reduced by the addition of high concentration (50 mM) of HCO_3_^–^ (Figure 1A). The results of the growth of the acetate-competent strain in these conditions showed that whatever the HCO_3_^–^ concentration, the growth started earlier (Figure 1B), indicating a decreased dependency of the growth start up to HCO_3_^–^ in the acetate-competent strain. Interestingly, the growth rate in the presence of 50 mM HCO_3_^–^ was significantly higher (*p* < 0.05) than in low bicarbonate conditions independently of the selected strain. The growth rate in low bicarbonate conditions was not significantly different between the two conditions (0 and 3 mM HCO_3_^–^) regardless of the strain. The observation that the early onset of the growth of the acetate-competent strain do not depend anymore on the addition of high concentration of HCO_3_^–^ suggests that this strain deals more efficiently with the light stress induced by the inoculation. This could imply that the acetate-competent strain more efficiently regulate intracellular redox imbalance. Indeed, we assume that the lag phase observed in the WT strain growing with acetate in low bicarbonate condition is due to a light-induced stress that is mainly characterized by a cellular redox stress. We attributed this redox stress to a massive reduction of the NAD^+^ pool due to an increased reverse flux through NADH dehydrogenase, explaining why added bicarbonate ions could help to balance this redox stress by being consumed through reduced equivalent-consuming pathways as previously described (Laguna et al., 2011; Mckinlay and Harwood, 2011; Gordon and McKinlay, 2014). The question is thus how does the acetate-competent strain more efficiently use the low level of available HCO_3_^–^ in the low bicarbonate conditions? Our hypothesis is that the already reported (De Meur et al., 2018) upregulation of the key enzyme of the EMC pathway [crotonyl-CoA carboxylase/reductase (Rru_A3063), ethylmalonyl-CoA mutase (Rru_A3062) and methylsuccinyl-CoA dehydrogenase (Rru_A3064)] in the acetate-competent strain increases the flux through the EMC pathway, thus consuming excess of reduced cofactors. The EMC pathway has already been proposed to play a role in redox homeostasis (Laguna et al., 2011).

### Metabolic Response to Light Stress in *Rs. rubrum* Growing in Various Carbon Source and Bicarbonate Condition

To deeper investigate how *Rs. rubrum* responds to the light-induced stress, we subjected the acetate-competent and the wild-type strains to a sudden increase in light intensity (from 50 μmol photons/m^2^ to 150 μmol photons/m^2^). This procedure will be referred to as light stress through this manuscript. To do so, whenever the OD_680nm_ reached ∼1.2, the light intensity was increased from 50 μmol photons/m^2^ s to 150 μmol photons/m^2^ s. To also take into account the effect of the assimilation of different carbon sources and the presence of bicarbonate ions on the response to the light stress, cultures were grown in either succinate or acetate and low or high bicarbonate condition (3 or 50 mM of HCO_3_^–^).

**FIGURE 2:**
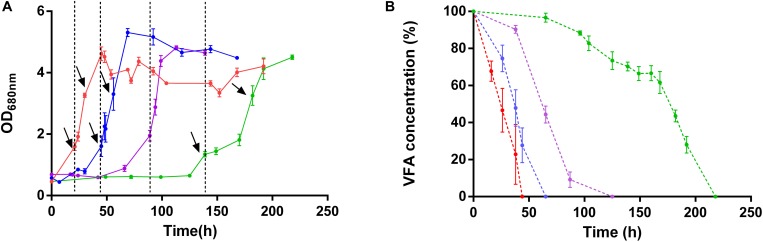
Growth profile **(A)** and carbon source consumption **(B)** of wild-type *Rs. rubrum* in the presence of succinate (red), acetate and 3 mM HCO_3_**^–^** (green) or 50 mM HCO_3_**^–^** (purple) and the acetate-competent strain (blue). Dotted black lines represent the increase in light intensity from 50 to 150 μmol of photons/m^2^ s. Black arrows represent the sampling time for the sample submitted to the proteomic analysis, *n* = 5.

As expected from the absence of the lag phase in this condition, the light stress had no effect on *Rs. rubrum* cultivated in the presence of succinate. In contrast, the wild-type strain grown with acetate as a sole carbon source in low bicarbonate condition exhibited a growth stop of approximately 24 h after the increase in light intensity (Figure 2A). This result suggests that the WT strain is more susceptible to the light stress when cultivated with acetate rather than succinate as the sole carbon source. The presence of 50 mM bicarbonate ions or the acclimation to acetate (acetate-competent strain) decreased this sensitivity since no stop in the growth profile was observed when light stress was applied in these conditions (Figure 2A). It is interesting to observe that the growth arrest in the WT strain growing on acetate in low bicarbonate condition was accompanied by a stop in acetate uptake (Figure 2B). This stop in acetate uptake suggests that the absence of growth is linked to an arrest of the metabolism. Intriguingly, WT strain cultivated in the presence of acetate in low bicarbonate condition not only presented a growth stop upon light stress but also showed impacted growth rate after growth restarted. Effectively, whereas the growth rates significantly (*p* < 0.05) increased after the increase in light intensity in all conditions (μ_Succinate_ = 0.058 ± 0.009; μ_Succinate after LS_ = 0.124 ± 0.035; μ_Acetate–competent strain_ = 0.054 ± 0.026; μ_*Acetate*−*competent* strain after LS_ = 0.139 ± 0.054; μ_Wild–type acetate 50 mM HCO3__–_ = 0.042 ± 0.005; μ_Wild–type acetate 50 mM HCO3 after LS__–_ = 0.093 ± 0.025; *p* < 0.05), the growth rates computed for WT with acetate in low bicarbonate condition showed no significant changes after the light increase (μ_Wild–type acetate 3 mM HCO3__–_ = 0.049 ± 0.009; μ_Wild–type acetate 3 mM HCO3 after LS__–_ = 0.037 ± 0.009). This result highlights the profound impacts on the metabolism in this condition and that WT strain was unable to take advantage of higher light intensity when cultivated with acetate under low bicarbonate condition. These results also suggested that the genomic amplification of a gene cluster that characterize the acetate-competent strain or the addition of high concentration of HCO_3_^–^ both permit a higher tolerance to high light intensity conditions.

### Proteomic Analysis of the Light Stress Response

**FIGURE 3:**
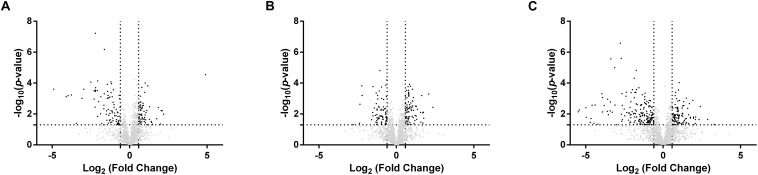
Volcano plots of the proteins quantified with significantly differential abundance between sample harvested before or after the light stress. The –log_10_(*p*-value) is plotted against the log_2_(fold change). The non-axial vertical lines denotes ± 1.5-fold change (prior to logarithmic transformation) while the non-axial horizontal line denotes *p* = 0.05, which is our significance threshold (prior to logarithmic transformation). **(A)** Wild-type strain cultivated with succinate (post light stress vs. pre light stress); **(B)** Acetate competent strain cultivated with acetate (post light stress vs. pre light stress); **(C)** Wild-type strain cultivated with acetate (post light stress vs. pre light stress).

To explore the response of *Rs. rubrum* in the different conditions subjected to light stress, we compared the response of the WT strain in succinate with the WT strain in acetate under low bicarbonate condition. To better understand the observed higher tolerance to the light stress in the acetate-competent strain, we also analyzed the response to the light stress at the proteomic level in this strain. For all three conditions, samples were taken just before the increase of the light intensity and immediately after the growth restarted. Sampling time in these different conditions is represented by black arrows in Figure 2A. In this analysis, 1824 proteins were identified and quantified, of which 1466 were identified with 2 or more peptides. The comparison of the proteome before and after the increase in the light intensity in the presence of succinate revealed that 316 proteins were statistically significantly impacted (either more or less abundant, *p* ≤ 0.05), while 337 and 387 proteins were significantly affected when cells were growing with acetate in the acetate-competent or WT strain, respectively. To select biologically relevant proteins, only proteins presenting a relative abundance lower than 0.66 or higher than 1.5 were considered for further analyzes. Therefore, 161 proteins were considered for the succinate condition, of which 86 presented a lower relative abundance and 75 showed a higher relative abundance after light intensity increase; 173 (70 with a lower relative abundance and 103 with a higher relative abundance after light intensity increase) for the acetate-competent strain and 290 (170 with a lower relative abundance and 120 with a higher relative abundance after light intensity increase) for the WT strain in the presence of acetate in low bicarbonate condition. In agreement with the phenotypic observations, when grown with acetate in low bicarbonate condition, the WT strain showed the strongest response at the proteomic level, as almost twice the number of proteins was significantly affected by the increase of the light intensity in comparison with the response in succinate (Figure 3).

**FIGURE 4:**
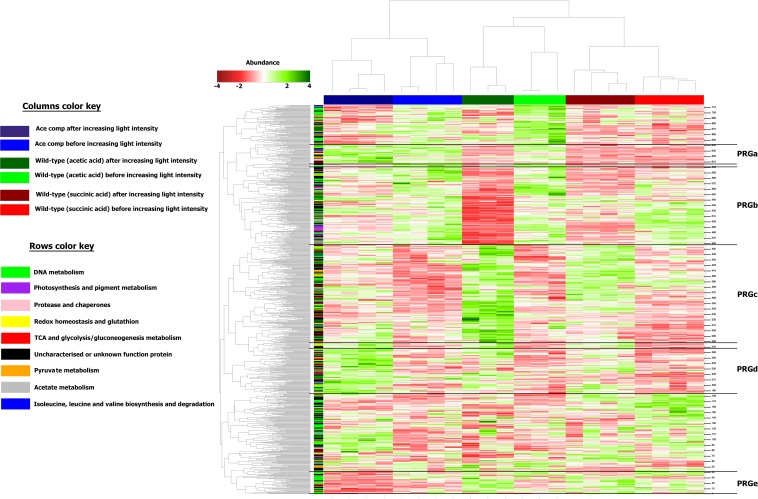
Heatmap representation of the differentially abundant proteins considered in this study. Columns were separated by using forced Euclidean Hierarchical Clustering based on the growth conditions. Rows were separated by unforced Euclidian hierarchical clustering. Protein response groups (PRGs) were established based on color pattern and supported by Monte-Carlo simulation.

Differentially regulated proteins were plotted in a heatmap (Figure 4). Based on Euclidian hierarchical clustering, we established 5 protein response groups (PRGs) corresponding to proteins whose abundance varied in the same way in response to the light stress (Supplementary Table S1). Among them, PRGa was composed of proteins characteristic of acetate metabolism meaning that they presented low abundance in the succinate condition and high abundance in all acetate conditions before the increase in light intensity. The abundance of those proteins decreased in the wild-type strain after the light stress, whereas their abundance in the acetate-competent strain remained stable or increased. In other words, the proteins of this PRG could explain how the acetate-competent strain keeps growing after the light stress.

PRGb is composed of proteins presenting a high abundance before the change in the light intensity and a drop in abundance after the increase in the light intensity. If the PRGb response applied to all conditions, the degree of the decrease of abundance was condition dependent. Indeed, if the decrease in the abundance of proteins of this PRG seems rather inexistent in the acetate-competent strain, we observed a dramatic decrease in the protein abundance in the WT strain cultivated in the presence of acetate in low bicarbonate condition. Thus, the proteins of this PRG could help to understand the global effect of increasing light intensity on *Rs. rubrum* and the exacerbated impact on the WT strain in the presence of acetate in low bicarbonate condition.

**FIGURE 5:**
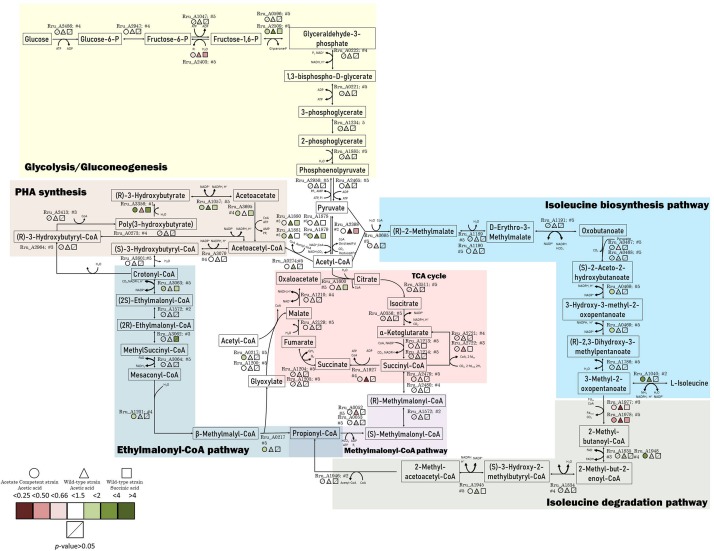
Schematic representation of the central carbon metabolism highlighted by the proteomic data. The colored markers represent the fold change, ranging from red (lower relative abundance) to green (higher relative abundance), of proteins identified and quantified with 2 or more peptides. Different conditions are represented by different forms. Stripped forms depict *p* > 0.05.

The proteins listed in PRGc presented a higher abundance after the light stress. However, the effect on WT strain cultivated in the presence of acetate in low bicarbonate condition seemed to be exacerbated, in contrast to the effect observed on the acetate-competent strain or in the WT strain in presence of succinate. Thus, this PRG contains proteins that could be considered as stress related proteins or as proteins that helped the WT strain to finally face the light stress in acetate condition. In contrast, PRGd was composed of proteins that were of low abundance in all three conditions before the light intensity increase and responded to the change in the light intensity by an increase in their abundance. This response was exacerbated in the acetate-competent strain, suggesting this PRG contains proteins that help this strain to face the change in light intensity. Finally, PRGe was composed of proteins that were decreased in terms of abundance in the acetate-competent strain (Figure 4). Complete proteomic data is available on supplementary data and metabolically relevant observations are represented in Figure 5 and Table 1.

**TABLE 1:** Differential abundance of selected (*p* ≤ 0.05; fold change ≤ 0.66 or ≥ 1.5) proteins.

Accession number	Locus tag	Identified peptides	Description	Acetate competent strain post vs. Acetate competent strain pre		WT strain acetate post vs. WT strain acetate pre		WT strain succinate post vs. WT strain succinate pre	
				*p*-value	Fold change	*p*-value	fold change	*p*-value	Fold change
**Photosynthesis machinery and pigment biosynthesis related proteins**									
Q2RYC8	Rru_A0062	3	Terpene synthase. squalene cyclase	0.28441	0.474	0.00111	0.084	7,25E-01	0.239
Q2RX47	Rru_A0493	4	Phytoene desaturase	0.86155	0.983	0.0163	0.600	0.02006	0.679
Q2RX46	Rru_A0494	3	Phytoene synthase	0.30362	0.759	0.00039	0.193	8,66E-01	0.176
Q2RWS6	Rru_A0615	4	Photosynthetic complex assembly protein	0.78745	0.962	0.01743	0.236	0.07089	0.781
Q2RWS5	Rru_A0616	5	Photosynthetic complex assembly protein	0.42155	0.854	0.01101	0.086	0.00378	0.499
Q2RWS2	Rru_A0619	5	Magnesium protoporphyrin O-methyltransferase	0.00116	0.565	0.00674	0.083	5,96E-04	0.216
Q2RWS1	Rru_A0620	5	Light-independent protochlorophyllide reductase iron-sulfur ATP-binding protein	0.00582	0.501	0.00049	0.065	0.00031	0.205
Q2RWS0	Rru_A0621	5	Hydrogenobyrinic acid a.c-diamide cobaltochelatase	0.03393	0.402	0.0821	0.106	0.0036	0.214
Q2RWR9	Rru_A0622	5	Light-independent protochlorophyllide reductase subunit B	0.00951	0.672	2,51E-02	0.153	0.00027	0.483
Q2RWR8	Rru_A0623	5	Light-independent protochlorophyllide reductase subunit N	0.03047	0.762	2,75E-02	0.096	0.04966	0.564
Q2RWR7	Rru_A0624	2	2-vinyl bacteriochlorophyllide hydratase	0.06832	0.473	0.01637	0.032	0.0002	0.218
Q2RWM0	Rru_A0671	5	Cobalamin synthesis CobW protein	0.02519	0.764	0.00233	0.475	0.26642	0.925
Q2RRE1	Rru_A2504	5	Coproporphyrinogen-III oxidase	0.18861	0.865	0.02198	0.478	0.00552	0.754
Q2RRD7	Rru_A2508	4	5-aminolevulinate synthase	0.00064	0.432	0.00397	0.032	0.00066	0.064
Q2RQ26	Rru_A2974	5	Photosynthetic reaction center M subunit	0.63765	0.923	0.08477	0.332	0.04142	0.596
Q2RQ23	Rru_A2977	2	Light-harvesting protein B-870 beta chain	0.1899	0.618	0.01463	0.358	0.01434	0.239
Q2RQ22	Rru_A2978	4	Chlorophyllide reductase subunit Z	0.00107	0.436	0.04608	0.054	0.00059	0.074
Q2RQ21	Rru_A2979	4	Chlorophyllide reductase subunit Y	0.00466	0.590	0.00048	0.159	0.00025	0.129
Q2RQ20	Rru_A2980	4	Chlorophyllide reductase iron protein subunit X	0.00017	0.351	0.00714	0.022	0.00077	0.058
Q2RQ19	Rru_A2981	5	2-desacetyl-2-hydroxyethyl bacteriochlorophyllide	0.45614	0.919	0.04329	0.369	0.01162	0.775
Q2RQ18	Rru_A2982	3	Hydroxyneurosporene-O-methyltransferase	0.06652	0.826	0.00478	0.418	0.48625	0.895
Q2RQ17	Rru_A2983	4	Farnesyl-diphosphate synthase	0.00018	0.514	0.0026	0.048	0.00025	0.034
Q2RNF3	Rru_A3548	5	Magnesium-protoporphyrin IX monomethyl ester anaerobic oxidative cyclase	0.17838	0.824	0.01129	0.434	0.02637	0.707
**Stress related proteins**									
Q2RYH0	Rru_A0020	4	Glutathione peroxidase	0.63415	1.078	0.00094	2.834	0.05436	1.654
Q2RXK8	Rru_A0332	5	Glutathione S-transferase-like	0.00062	1.738	0.01642	2.012	0.00345	1.420
Q2RX78	Rru_A0462	5	Aldehyde dehydrogenase	0.04422	1.622	0.00214	3.251	0.00345	1.330
Q2RX66	Rru_A0474	5	Zinc-containing alcohol dehydrogenase superfamily	0.0305	1.317	0.01699	1.305	0.0027	1.600
Q2RU91	Rru_A1503	4	Zinc-containing alcohol dehydrogenase superfamily	0.21166	1.539	0.09942	1.566	0.00456	1.713
Q2RNU6	Rru_A3405	4	Formaldehyde dehydrogenase (Glutathione)	0.02883	1.587	0.01313	2.288	0.02845	1.604
**Calvin-benson-bassham cycle related proteins**									
Q2RRP5	Rru_A2400	5	Ribulose bisphosphate carboxylase	0.03871	0.509	0.41425	1.236	0.30792	0.863
**Sugar metabolism related proteins**									
Q2RT67	Rru_A1878	5	Dihydrolipoyl dehydrogenase	0.07458	1.350	0.00146	1.941	0.01495	1.422
Q2RT66	Rru_A1879	5	Acetyltransferase component of pyruvate dehydrogenase complex	0.00017	1.689	0.02696	2.590	0.00243	1.480
Q2RT65	Rru_A1880	5	Pyruvate dehydrogenase beta subunit	0.00118	1.451	0.00133	2.093	0.00418	1.516
Q2RT64	Rru_A1881	5	Pyruvate dehydrogenase E1 component subunit alpha	0.00071	1.742	0.0122	2.017	0.01036	1.418
Q53046	Rru_A2398	2	Pyruvate-flavodoxin oxidoreductase	0.03605	0.783	0.01965	0.040	0.00275	0.318
Q2RRP2	Rru_A2403	5	Fructose-1.6-bisphosphatase class 1	0.01406	0.503	0.00885	0.376	0.02643	0.477
**ILV BIosynthesis pathway related proteins**									
Q2RX73	Rru_A0467	5	Acetolactate synthase. large subunit	0.07267	1.122	0.0261	0.586	0.15251	1.281
Q2RVK4	Rru_A1040	2	Leucine dehydrogenase	0.01376	2.430	0.01115	3.139	0.15537	1.158
Q2RSW8	Rru_A1977	3	Pyruvate ferredoxin/flavodoxin oxidoreductase	0.02913	0.619	1,01E-01	0.115	0.03514	0.605
Q2RSW7	Rru_A1978	5	Indolepyruvate oxidoreductase subunit IorA	0.00386	0.462	0.00629	0.094	0.00011	0.445
Q53046	Rru_A2398	2	Pyruvate-flavodoxin oxidoreductase	0.03605	0.783	0.01965	0.040	0.00275	0.318
**EMC Pathway related proteins**									
Q2RV43	Rru_A1201	4	Malyl-CoA/β-methylmalyl-CoA lyase	0.00051	1.764	0.30914	0.827	0.98208	0.996
Q2RPT7	Rru_A3063	5	Crotonyl-CoA carboxylase/reductase	0.1422	1.792	0.13806	0.637	0.03048	1.929
**PHA Production related proteins**									
Q2RXR4	Rru_A0276	5	Polyhydroxyalkanoate synthesis repressor PhaR	0.47385	0.947	0.01299	0.436	0.49704	0.932
Q2RVI7	Rru_A1057	5	3-hydroxybutyrate dehydrogenase	0.0021	1.508	0.00604	1.968	0.00071	1.630
Q2RNZ5	Rru_A3356	1	Polyhydroxyalkanoate depolymerase	0.00849	3.279	0.00971	0.267	0.06107	2.404
Q2RN06	Rru_A3695	4	Acetoacetyl-CoA synthase	0.00584	1.518	0.23529	1.191	0.00295	1.766
**Acetate-competent duplication phenomenon related proteins**									
Q2RPR7	Rru_A3083	5	NADPH-dependent FMN reductase	0.08552	1.298	0.03889	2.010	0.017	1.754

#### Effect of Light Stress on Photosynthesis Machinery

Several proteins related to photosynthesis mechanisms or pigment biosynthesis were observed with a modified abundance after light intensity increase (Table 1). This type of result was already described by Niederman’s group in purple bacteria (Niederman, 2013) or in the unicellular chlorophyte alga, *Dunaliella tertiolecta*, by Escoubas et al. (1995). Indeed, a lower abundance of the photosynthetic-related proteins was observed after the increase in light intensity in all conditions. However, the wild-type strain cultivated in the presence of acetate in low bicarbonate condition constitutes the most affected condition by high light intensity. This large amount of regulated proteins related to photosynthesis and pigment biosynthesis constitutes a demonstration of the stress induced by the increase in light intensity. A reduced abundance of photosynthetic proteins can be explained by the need for the bacterium to reduce photon capture and the resulting generation of a too large proton gradient which could lead to an increased photoreduction of NAD^+^ into NADH. Limitation of light harvesting capacity could also prevent the production of overexcited bacteriochlorophyll leading to the photooxidation phenomenon described previously (Glaeser and Klug, 2005; Hallenbeck, 2010; Razjivin et al., 2018).

#### Effect of the Light Stress on Stress Related Proteins

As expected, stress-related proteins such as glutathione peroxidase (Rru_A0020) showed significantly higher relative abundance in both wild-type conditions (WT acetate: *p* = 9.40E-04; fold change = 2.83 and WT succinate: *p* = 0.05; fold change = 1.65). Interestingly this protein was not upregulated in the acetate-competent strain which could reflect the lower stress triggered by the light intensity increase in this strain. Another redox-related protein was found more abundant after the light stress in acetate growing cultures, the glutathione-S-transferases (Rru_A0332). Several proteins belonging to the reactive oxygen species (ROS) detox system, such as aldehyde dehydrogenase, two zinc-containing alcohol dehydrogenases and a formaldehyde dehydrogenase, using glutathione showed significantly impacted abundance. Aldehyde dehydrogenases are involved in a variety of biological processes in prokaryotes. Their expression is upregulated in response to abiotic or biotic stress. These proteins are known to decrease ROS stress caused by aldehydes, which can be produced when bacteria are exposed to heat, dehydration or oxidants. Moreover, there is evidence that this family of proteins is produced as a critical component to face environmental stresses, particularly the oxidative stress response (Singh et al., 2013). Taken together, the data obtained for these stress related proteins reflected an intracellular detoxification phenomenon showing evidences of a cellular stress induced by the increase in the light intensity. Moreover, this phenomenon was more obvious in the WT strain cultivated in the presence of acetate in low bicarbonate condition. It seems again that, somehow, the acetate-competent strain is able to maintain the stress at a lower level. Additionally, our results corroborate those obtained by Billenkamp et al. (2015), who indicated that photooxidative stress (e.g., the induction of ROS production by photosynthetic processes) induces a redox signal in *Rhodobacter sphaeroides*, leading to the expression of the formaldehyde detoxification pathway.

#### Effect of Light Stress on Calvin-Benson-Bassham Cycle

The ribulose bisphosphate carboxylase (Rru_A2400), implicated in the Calvin-Benson-Bassham cycle and necessary for the assimilation of CO_2_ by this cycle, showed a significantly lower abundance in the acetate-competent strain after the increase in the light intensity (Rru_A2400; acetate competent: *p* = 0.04, fold change = 0.51; WT acetate: *p* = 0.41, fold change = 1.24; WT succinate: *p* = 0.31, fold change = 0.86). We already observed that under acetate growing conditions, RuBisCO was downregulated in comparison to succinate condition in *Rs. rubrum*. The possible explanation is that the EMC pathway is used in acetate growing cells as a CO_2_ assimilation and redox balancing pathway (Anthony, 2011; Bill et al., 2017). However, why the downregulation of RuBisCO could effectively be beneficial for the adaptation to the sudden light increase is still unclear today and would require further investigations.

#### Effect of Light Stress on Sugar Metabolism

The four subunits of the pyruvate dehydrogenase (dihydrolipoyl dehydrogenase Rru_A1878, acetyltransferase component of pyruvate dehydrogenase complex Rru_A1879, pyruvate dehydrogenase alpha, and beta subunits Rru_A1880-81) were all observed to be more abundant in WT cultivated with acetate in low bicarbonate condition, while they were only partially regulated in other conditions (Table 1 and Figure 5). This result could be linked to the observation of a lower relative abundance, in the WT strain cultivated in acetate, of the PFOR (Rru_A2398), which catalyzes the reverse reaction (McNeely et al., 2011). As pyruvate is a key metabolite of the ILV biosynthesis, this could reflect the inability of the WT strain to use this key pathway in response to light stress when growing with acetate, explaining lower tolerance to light increase in this condition.

**FIGURE 6:**
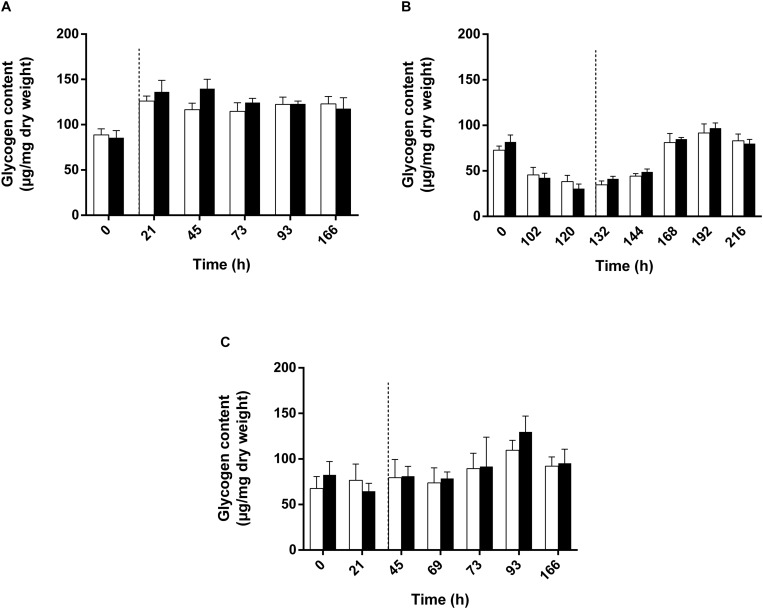
Polysaccharide quantification of WT *Rs. rubrum* cultivated with succinate **(A)**, acetate and 3 mM HCO_3_^–^ **(B)** or the acetate-competent strain cultivated with acetate **(C)**. Uncolored bars represent the condition without any light stress; dark colored bars represent the condition subjected to the light stress. Dotted black lines represent the increase in light intensity from 50 to 150 μmol of photons/m^2^ s.

The observation of a higher relative abundance of pyruvate dehydrogenase could indicate a higher mobilization of glycogen through glycolysis. Observation of the downregulation of fructose-1,6-bisphosphatase in all conditions also supported this hypothesis (Table 1). Glycogen constitutes a major intracellular carbon reserve consisting of α-1,4-linked glucose. In bacteria, this polymer accumulates during limiting growth conditions when an excess of a carbon source is available and several studies have already linked glycogen metabolism to environmental survival. In order to test whether glycogen was mobilized during adaptation to high light intensity, we measured the polysaccharide content of the biomass in cultures subjected or not to a sudden increase of the light intensity. Surprisingly, the polysaccharide content did not seem to be impacted by the light stress. Indeed, no significant difference was observed between conditions where the cells were not subjected to light stress and those that were subjected to the increase of the light intensity (Figure 6). Regardless of the light regime, a higher sugar content was found in the presence of succinate, reaching 150 μg/mg dry weight (Figure 6A), while the polysaccharide content reached 100 μg/mg dry weight in the presence of acetate (Figures 6B,C). Interestingly, in the WT strain cultivated with acetate in low bicarbonate conditions, the polysaccharide content significantly decreased during the lag phase before increasing during the exponential phase (Figure 6B). This means that the lag phase characteristic of the growth with acetate in low bicarbonate condition is also characterized by the mobilization of internal stock of sugar by the cell acting as a source of pyruvate/PEP. Therefore, it may substitute the EMC pathway as anaplerotic pathway sustaining the survivability of the bacteria. Interestingly no such mobilization of glycogen could be observed in the acetate-competent strain, reinforcing the hypothesis of the better adaptation of this strain to the assimilation of acetate.

#### Effect of the Light Stress on ILV Biosynthesis Pathway

The ILV biosynthesis pathway was already proposed as being potentially involved in acetate metabolism in previous studies conducted by our group (Leroy et al., 2015; De Meur et al., 2018). More recently, we proposed that this pathway could be used as a new assimilatory pathway in *Rs. rubrum* in the context of butyrate assimilation (De Meur et al., submitted).

The pyruvate flavodoxin/ferredoxin oxidoreductase (PFOR) showed a massively lower relative abundance after the increase in light intensity in the WT strain growing with acetate (*p* = 0.02; fold change = 0.04) and a lower abundance in the WT with succinate (*p* = 2.75E-03; fold change = 0.32). On the other hand, the fold change was not significant in the acetate-competent strain. This enzyme catalyzes the reductive carboxylation of acetyl-CoA into pyruvate in anaerobic organisms cultivated in the presence of acetate (St. Maurice et al., 2007), and has been proposed by our group to be involved in acetate assimilation in *Rs. rubrum* (Leroy et al., 2015). The fact that the abundance of this enzyme is maintained in acetate growing cells that tolerate the light increase (namely the acetate-competent strain) while it almost totally disappeared from cell that could not tolerate the stress (WT with acetate) reinforce the importance of this enzyme for acetate assimilation.

In this study, the large subunit of acetolactate synthase presented a lower relative abundance after the light stress (Rru_A0467, *p* = 0.03; fold change = 0.59) in the WT strain with acetate in low bicarbonate condition, while no differences were observed in the two other conditions. This enzyme is involved in the entry of pyruvate in the ILV biosynthesis pathway.

Our data also revealed that the leucine dehydrogenase (Rru_A1040), the enzyme catalyzing the final step of the ILV biosynthesis pathway, was upregulated after the light stress in the cultures growing with acetate (both WT and acetate-competent strains). Our group already highlighted the potential involvement of ILV biosynthesis in the metabolism of VFAs in *Rs. rubrum* (Leroy et al., 2015; De Meur et al., 2018, submitted). This enzyme showed higher relative abundance in acetate conditions after the increase in the light intensity change (acetate-competent: *p* = 0.01; fold change = 2.43; wild-type acetate: *p* = 0.01; fold change = 3.14). Starting from acetyl-CoA, biosynthesis of ILV consumes 2 reduced ferredoxin and 2 NADPH (Figure 5), globally representing a way to maintain cellular redox potential (Shimizu et al., 2010; McCully et al., 2020). We recently proposed that enzymes of the ILV biosynthesis pathway combined with enzymes of the isoleucine degradation pathway could represent a new assimilation route for VFAs (De Meur et al., submitted). In this assimilatory pathway, Rru_A1977 and Rru_A1978, which belong to the oxoacid oxidoreductase family, were proposed to bridge ILV biosynthesis and isoleucine degradation pathways. Very interestingly, these enzymes were downregulated in all conditions after light stress, suggesting the shutdown of the flux through isoleucine degradation route. This result suggests that ILV or one of the intermediary metabolite of their synthesis could be, at least transiently, produced only in order to consume reducing power. To test this hypothesis, we analyzed the accumulation of the branched chain amino acids (ILV) in the culture medium. Absence of BCAAs in the culture medium (LOD < 10 μM) indicated that BCAAs were not excreted (data not shown). BCCAs could anyway be accumulated, probably transiently, intracellularly. Indeed, a recent study showed that the synthesis of ILV is impacted by the alarmone system which responds to environmental changes such as nutrient availability, temperature or dehydratation. In that context, bacteria are known to regulate the transcription of ILV biosynthesis genes in response to alarmone level (Gaca et al., 2015; Hauryliuk et al., 2015; Fang and Bauer, 2018) and high level of ILV have notably be detected in *Rh. sphaeroides* in that context. Further experiments will be required to test whether BCAAs could be produced and transiently accumulated intracellularly in order to help equilibrating redox balance in response to a light stress.

#### Effect of the Light Stress on the Enzymes of the EMC Pathway

The mesaconyl-CoA hydratase (Rru_A1201) and malyl-CoA/β-methylmalyl-CoA lyase were found to be more abundant only in the acetate-competent strain (Table 1 and Figure 5). These enzymes are known to belong to the EMC pathway. The EMC pathway, which is proposed to be the main pathway used for acetate assimilation (Leroy et al., 2015; De Meur et al., 2018), seems thus to be maintained in the WT starting growing with acetate and even increased in the acetate-competent strain. As this pathway consumes reducing equivalent, maintenance of a high flux in this pathway could be a key element in the adaptation to the sudden light increase. However, regulation observed here was of limited amplitude and only concerned few enzymes of the pathway. It is important to mention here that three enzymes of the EMC pathway and another one allowing acetyl-CoA to enter this EMC pathway are coded by the genomic region which was shown to be amplified in the acetate-competent strain [Rru_A3062: ethylmalonyl-CoA mutase; Rru_A3063: crotonyl-CoA carboxylase/reductase; Rru_A3064: (2*S*)-methylsuccinyl-CoA dehydrogenase; Rru_A3079: (*S*)-3-hydroxybutyryl-CoA dehydrogenase, see Figure 5]. Even if these enzymes do not seem to be significantly upregulated in response to the light stress, their abundance was higher in acetate-competent strain than in WT strain growing with acetate before the light stress. Their higher abundance is exacerbated after the light stress suggesting higher flux in the EMC pathway could be part of the adaptive response of the acetate-competent strain (Supplementary Table S2). A flux analysis should be performed in order to demonstrate whether the flux through the EMC pathway is effectively increased in response to light stress.

Very interestingly, the crotonyl-CoA carboxylase/reductase (Rru_A3063), one of the key enzymes of the EMC pathway, was found to be more abundant after the light stress in the succinate condition and acetate-competent strain (Rru_A3063_SuccPost/SuccPre_: *p* = 0.03; fold change = 1.93; Rru_A3063_AcecompPost/AcecompPre_: *p* = 0.14; fold change = 1.79) even if the significance criterion was not reached for the latter (Figure 5).

Since now, this key enzyme of the EMC pathway has always been linked to the presence and assimilation of acetate (Alber, 2011; Anthony, 2011; Schneider et al., 2012; Fuchs and Berg, 2014; Leroy et al., 2015; De Meur et al., 2018). A higher abundance of crotonyl-CoA carboxylase/reductase after the change in light intensity in the presence of succinate raised the question of the potential implication of the EMC pathway, and of the crotonyl-CoA carboxylase/reductase in particular, in the tolerance of *Rs. rubrum* to the light stress and in the redox homeostasis in general. In order to test the requirement of the crotonyl-CoA carboxylase/reductase for *Rs. rubrum* in the tolerance to a light intensity increase under succinate condition, we submitted our Δ*ccr* mutant growing with succinate to the light stress. The results of this experiment demonstrated that the crotonyl-CoA carboxylase/reductase was not essential for tolerance to the light stress in the presence of succinate in the experimental conditions of this study (see Supplementary Figure S2). It would be interesting to test the tolerance of our WT and Δ*ccr* strains to higher amplitudes of light stress since requirement of crotonyl-CoA carboxylase/reductase may only appears at higher light intensities.

#### Effect of the Light Stress on PHA Production

**FIGURE 7:**
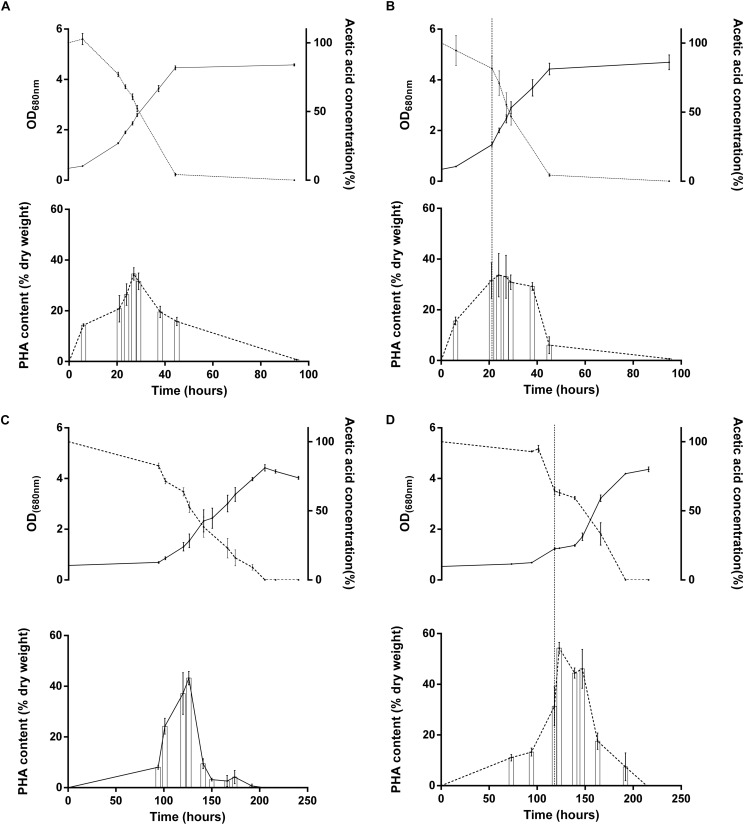
Growth (full line; upper panel), acetate consumption (dotted line, upper panel) and PHA accumulation (lower panel) of *Rs. rubrum* for the acetate-competent strain **(A,B)** or Wild type strain **(C,D)** in presence of acetate supplemented with 3 mM HCO_3_^–^ submitted **(B,D)** or not **(A,C)** to a light intensity increase from 50 to 150 μmol photons/m^2^ s. Vertical dotted line represents the light stress **(B,D)** *N* = 5.

We observed that multiple proteins related to PHA production were affected by the light stress. This hypothesis makes sense considering that PHA synthesis has already been reported to be activated in case of redox imbalance (Hauf et al., 2013). The polyhydroxyalkanoate (PHAs) synthesis repressor PhaR which act as a repressor of the synthesis of PHAs and PHA granule formation in a wide range of bacteria (Pfeiffer and Jendrossek, 2011; Wang et al., 2011; Cai et al., 2015; Quelas et al., 2016) was observed in lower relative abundance in the case of WT acetate after light increase (*p* = 0.01; fold change = 0.44). This could reveal an increased production of PHAs in order to equilibrate redox balance in this condition. Additionally, we also highlighted the higher relative abundance of the 3-hydroxybutyrate dehydrogenase (Rru_A1057), which catalyzes the reversible conversion of acetoacetate into 3-hydroxybutyrate, in the WT strain under acetate condition and to a lower extent in the two other conditions. Interestingly, the acetoacetyl-CoA synthetase (Rru_A3695), which catalyzes the irreversible conversion of acetoacetate into acetoacetyl-CoA, was slightly up in acetate-competent strain growing with acetate and WT strain growing with succinate but not in WT strain growing with acetate. This enzyme is implicated in PHA mobilization (Figure 5). Similarly, the polyhydroxyalkanoate depolymerase (Rru_A3356, quantified here with only one single peptide) was highly upregulated (*p-*value: 0.008, fold change: 3.28) in acetate-competent strain and largely downregulated (*p*-value: 0.01, fold change: 0.27) in WT strain growing with acetate. These results suggest that PHA production could be used differently by WT and acetate-competent strains to adapt the light stress. As PHA production constitutes a potential electron sink (Hauf et al., 2013) and as the regulation of proteins involved in their production suggest it could be involved in differential response to light stress between acetate-competent and WT strains, we compared PHA content observed in both strains subjected or not to light stress. PHA content quantitation revealed that the acetate-competent strain is able to accumulate up to 34.53 ± 3.67% of its dry weight as PHB under 50 μmol photon/m^2^ s. This maximal production was observed after 27 h of culture (Figure 7A). No significant difference could be observed (*p >* 0.05) for the acetate competent strain subjected to the light intensity increase (33.68 ± 8.14% of PHB accumulation) (Figure 7B). For the WT strain cultivated at 50 μmol photons/m^2^ s, maximum accumulation was observed after 126 h corresponding once again to the mid exponential phase (Figure 7C). Under those conditions, *Rs. rubrum* was able to accumulate up to 43.21 ± 3.78% of its dry weight as PHB. PHB content is significantly higher than those observed for the acetate competent strain (*p* < 0.05). In addition, in the case of the WT strain cultivated with acetate in low bicarbonate condition, the light stress was shown to induce a significant increase (*p* < 0.05) of the PHA content, reaching 54.29 ± 3.45% of the dry weight as PHB. It represents a rise of 25.64% of the PHB content (Figure 7D). This PHB accumulation was observed only 5 h after the increase of light intensity. These results suggest that the acetate-competent strain, being adapted to acetate assimilation make a lower use of the PHA synthesis as an electron sink even when a light stress is applied. On the other hand, in the WT strain growing with acetate in low carbonate condition, the cells accumulated a larger amount of PHA, probably reflecting higher redox imbalance. We here demonstrated that the light stress reinforced this accumulation, suggesting that the sudden increase of light intensity effectively increase the redox imbalance in acetate growing *Rs. rubrum*.

#### Impact of Genetic Background of Acetate-Competent Strain on Tolerance to Light Stress

As already mentioned the acetate-competent strain is characterized by the amplification of a genomic region ranging from Rru_A3000 to Rru_3120 and centered on three genes from the EMC pathway, in particular the crotonyl-CoA carboxylase/reductase, the implication of which has already been discussed. Analyzing the global response of the proteins coded by this genomic region, it clearly appeared that several of these proteins were in higher abundances before the light stress in the acetate-competent strain compared to the WT strain. In addition, this higher abundance was exacerbated by the light stress, notably for the genomic region ranging from Rru_A3062 to Rru_A3105, where all but two of the quantified proteins were significantly upregulated between acetate-competent and WT strain after the light stress (Supplementary Table S2).

Among the proteins encoded in this region, the NADH-FMN reductase (Rru_A3083) was observed with higher abundance after the light increase in WT growing with succinate and acetate but not in the acetate-competent strain. This observation suggests that this FMN reductase could serve as a redox balancing enzyme useful to adapt to the stress induced by the light intensity increase. When looking at the fold change of this enzyme in the acetate-competent strain, it did not seem to be regulated in this condition. However, the abundance of this protein was already very high in the acetate-competent strain before the increase of the light intensity. Indeed, comparing abundance of this enzyme in acetate-competent strain and WT strain growing with acetate, we observed that this protein was constitutively 2.82 times more abundant in acetate-competent strain (*p* = 1.10E-05). This indicate that this enzyme could be part of the genetic background advantage that the gene amplification provides to the acetate-competent strain and could be a major actor in adaptation to acetate condition and high light induced stress.

No other proteins belonging to the amplified genomic region could be detected with regulated abundance in individual condition in response to the light stress. Anyway, some elements from this genomic region could participate in the tolerance to light stress observed in the acetate-competent strain. Indeed, five transcriptional regulators were detected within the amplified region (Rru_A3003, Rru_A3041, Rru_A3065, Rru_A3067, and Rru_A3082). The targets of those regulators are not known yet but could take part in the light intensity resistance of this strain. Moreover, the amplified region also comprises a NAD(P)H dehydrogenase (quinone) (Rru_A3066). This enzyme could take part in the tolerance of the acetate-competent strain. Indeed, NAD(P)H dehydrogenases (quinone) are known to avoid the overreduction of the quinone pool during cyclic photosynthesis. It couples the reduction of NAD(P)^+^ to the oxidation of quinol, generating NAD(P)H. The enzyme would generate large amount of reduced cofactors that would have to be dissipated through metabolic systems. However, this enzyme could not be detected in our proteomic analysis.

## Conclusion

In conclusion, our results demonstrated that a high level of bicarbonate ions facilitated the onset of growth and drastically reduced the lag phase duration when acetate is used as the sole carbon source. A high level of bicarbonate ions was also able to increase the tolerance of *Rs. rubrum* to a sudden increase in light intensity. A higher tolerance to light stress, at inoculation or upon a sudden increase in the light intensity, was also observed for our acetate-competent strain even in low bicarbonate conditions. The prevalent hypothesis is that both the high level of bicarbonate ions and the genomic amplification in the acetate-competent strain help in dealing with the redox imbalance produced by the culture dilution at inoculation or a sudden light intensity increase. Our experiments demonstrated that high light intensity impacted photosynthesis and pigment metabolisms, confirming the findings of other groups (Carius et al., 2011; Niederman, 2013). Furthermore, the light stress also influenced the central carbon metabolism of *Rs. rubrum* and proteins implicated in redox homeostasis, indicating that increasing light intensity induced both photooxidative stress and intracellular redox stress especially in the WT strain cultivated in presence of acetate in low bicarbonate condition. Among the genes present in the genomic region which is amplified in the acetate-competent strain, only few were effectively responding to the light stress. A NADPH dependent FMN reductase was identified as a potential explanation of the light stress tolerance of this strain, but involvement of other elements of this genomic region cannot be ruled out. Our results also pointed toward the possible involvement of ILV biosynthesis as a redox balancing mechanism. However, as we could not detect any accumulation of BCAAs in the culture medium this hypothesis will require further investigations. We finally demonstrated that PHA accumulation, probably acting as an electron sink, was also used by the cell to adapt to a sudden increase of the light intensity. This response was only observed in the WT strain growing with acetate in low bicarbonate ions condition and not in the acetate-competent strain. This finding has important implication for the biotechnological use of purple bacteria in the context of bioplastic production since (i) we showed that long term cultivation of *Rs. rubrum* could lead to genetic adaptation and decrease in the PHA production efficiency and (ii) sudden light increase could be a way to induce redox stress in culture and force higher PHA accumulation.

## Data Availability Statement

The datasets generated for this study can be found in the https://db.systemsbiology.net/sbeams/cgi/PeptideAtlas/PASS_View?identifier=PASS01435.

## Author Contributions

GB-V, RW, and BL designed the study. GB-V performed the *Rs. rubrum* cultivations experiments, proteomic analysis, and the bioinformatic analysis. GB-V and BL designed the proteomic analysis. GB-V wrote the manuscript with the help of BL and RW.

## Conflict of Interest

The authors declare that the research was conducted in the absence of any commercial or financial relationships that could be construed as a potential conflict of interest.

## Footnotes

1 https://db.systemsbiology.net/sbeams/cgi/PeptideAtlas/PASS_View?identifier=PASS01435

## Supplementary Material

FIGURE S1 Monte-Carlo simulation confirming robustness of the Heatmap.

FIGURE S2 Growth of *Rs. rubrum* wild type (red curve) and ΔCcr (green and blue curves) strain cultivated with succinate as sole source of carbon subjected to a increasing light intensity (150 μmol photons/m^2^ s) (red and blue curves) or to constant 50 μmol of photons/m^2^ s. Dotted line depicts the increase of light intensity.

TABLE S1 HeatMap related proteins presenting *p*-value < 0.05.

TABLE S2 Duplication phenomenon related proteins identified during proteomic analysis.
