# Supplementary figures and images for: Global Proteomic Analysis Reveals High Light Intensity Adaptation Strategies and Polyhydroxyalkanoate Production in Rhodospirillum rubrum Cultivated With Acetate as Carbon Source
Source: Front Microbiol. 2020 Mar 25;11:464. doi: 10.3389/fmicb.2020.00464 (PMC7109303; doi:10.3389/fmicb.2020.00464)

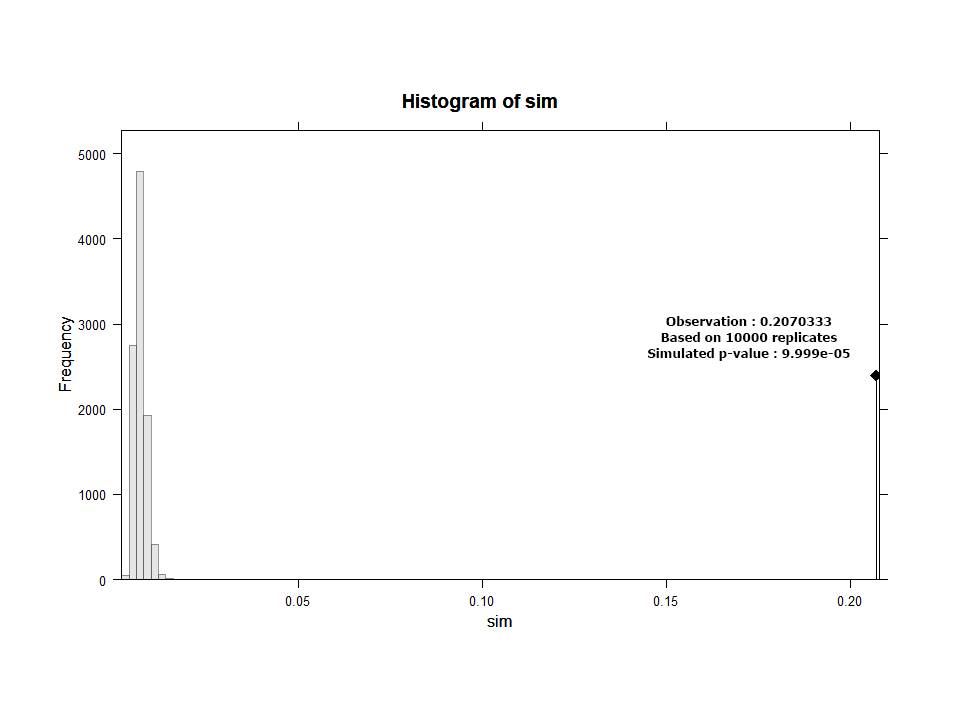

Supplement: FIGURE S1 — Monte-Carlo simulation confirming robustness of the Heatmap. [file Image_1.JPEG]

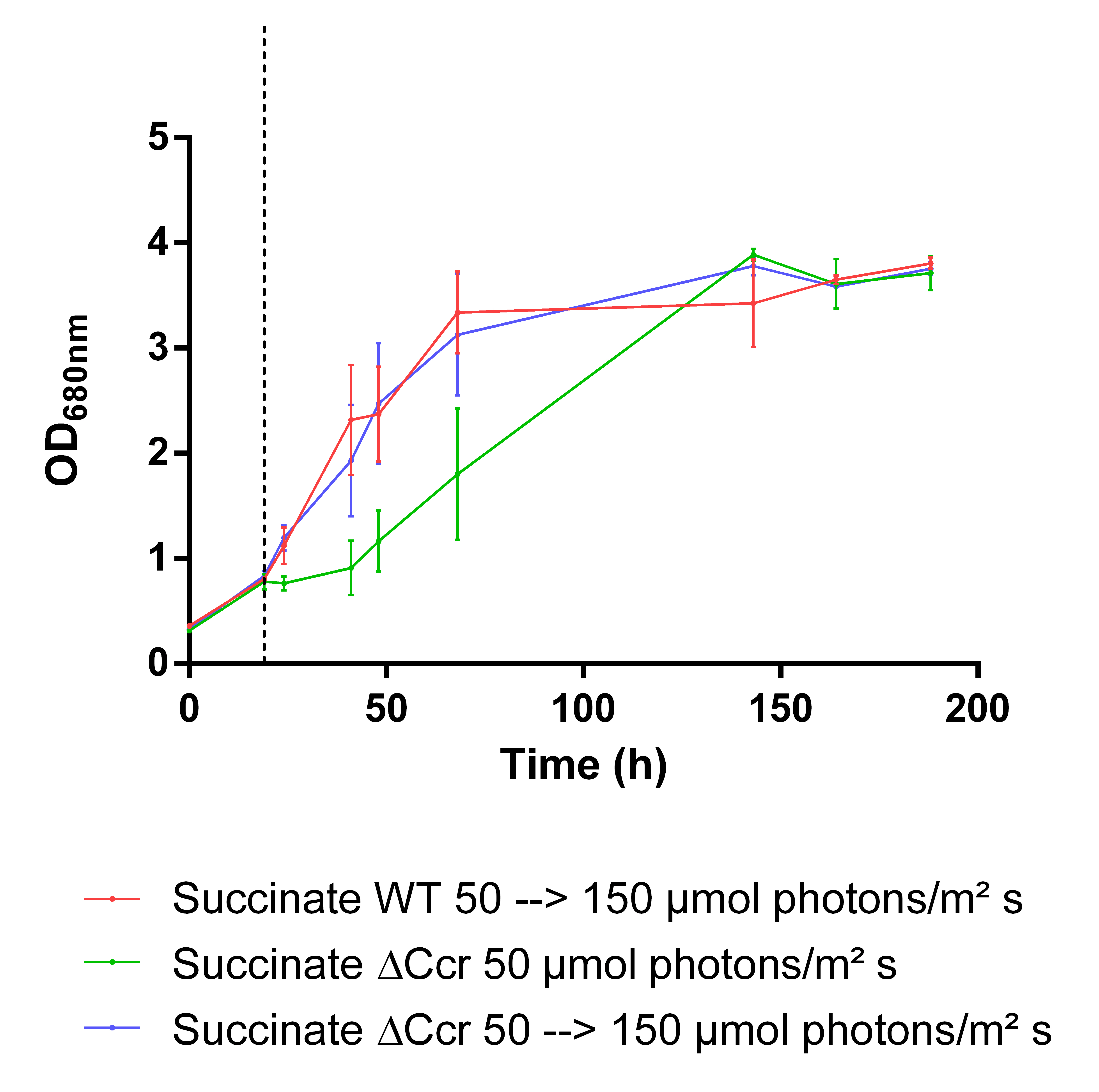

Supplement: FIGURE S2 — Growth of Rs. rubrum wild type (red curve) and ΔCcr (green and blue curves) strain cultivated with succinate as sole source of carbon subjected to a increasing light intensity (150 μmol photons/m2 s) (red and blue curves) or to constant 50 μmol of photons/m2 s. Dotted line depicts the increase of light intensity. [file Image_2.JPEG]
